# “Total reconstruction” of the urethrovesical anastomosis contributes to early urinary continence in laparoscopic radical prostatectomy

**DOI:** 10.1590/S1677-5538.IBJU.2014.0666

**Published:** 2016

**Authors:** Xiaoxing Liao, Peng Qiao, Zhaohui Tan, Hongbin Shi, Nianzeng Xing

**Affiliations:** 1 Department of Urology, Beijing Chao-Yang Hospital, Capital Medical University, Beijing, China; 2 Department of Urology, Beijing Aerospace General Hospital, Beijing, China; 3 Department of Urology, Inner Mongolia People's Hospital, Inner Mongolia, China; 4 Department of Urology, General Hospital of Ningxia Medical University, Yinchuan, China

**Keywords:** Laparoscopy, Prostatectomy, Urinary Incontinence, Prostatic Neoplasms, Reconstructive Surgical Procedures

## Abstract

**Purpose:**

To demonstrate the effect of total reconstruction technique on postoperative urinary continence after laparoscopic radical prostatectomy (LRP).

**Material and Methods:**

LRP was performed using a standard urethrovesical anastomosis in 79 consecutive patients (Group-A) from June 2011 to October 2012, and a total reconstruction procedure in 82 consecutive patients (Group-B) from June 2012 to June 2013. The primary outcome measurement was urinary continence assessed at 1, 2, 4, 12, 24 and 52 weeks after catheter removal. Other data recorded were patient age, body mass index, International Prostate Symptoms Score, prostate volume, preoperative PSA, Gleason score, neurovascular bundle preservation, operation time, estimated blood loss, complications and pathology results.

**Results:**

In Group-A, the continence rates at 1, 2, 4, 12, 24 and 52 weeks were 7.59%, 20.25%, 37.97%, 58.22%, 81.01% and 89.87% respectively. In Group-B, the continence rates were 13.41%, 32.92%, 65.85%, 81.71%, 90.24% and 95.12% respectively. Group––B had significantly higher continence rates at 4 and 12 weeks after surgery (P<0.001 and P=0.001). There were no significant differences between the groups with respect to patient's age, body mass index, prostate-specific antigen level, prostate volume, IPSS, estimated blood loss, number of nerve-sparing procedures and postoperative complications.

**Conclusions:**

Total reconstruction technique in the procedure of urethrovesical anastomosis during LRP improved early recovery of continence.

## INTRODUCTION

Radical prostatectomy (RP) is the standard surgical treatment for localized prostate cancer (1). Postoperative urinary incontinence is one of the drawbacks after RP, especially early incontinence, which has a major impact on patient's health-related quality of life (2). Post-prostatectomy incontinence has been attributed to damage to the arterial supply of the vesical sphincter, nerve and the integrity of the pelvic floor muscles (3). Several surgical technical modifications have been proposed to minimize the incidence of urinary incontinence, including nerve sparing (4), bladder neck preservation (5), sparing or reconstruction of the puboprostatic ligament (6–8), posterior reconstruction of the rhabdomyosphincter (9, 10), and anterior retropubic suspension (11, 12). These techniques are associated with different improvement on early continence. More studies on surgical modifications are still needed to improve postoperative early continence.

In an effort to improve early urinary continence, we applied a total reconstruction technique during laparoscopic radical prostatectomy (LRP). In the present study, we compared the perioperative and urinary continence outcomes of LRP with and without total reconstruction technique.

## MATERIALS AND METHODS

From June 2011 to June 2013, a total of 161 consecutive patients that underwent LRP were reviewed, of which 79 patients (Group-A) were treated with standard anastomosis technique from June 2011 to October 2012, and 82 patients (Group-B) were managed with total reconstruction technique from June 2012 to June 2013. This study was approved by the Ethical Committee of our hospital. All surgeries were performed by a single surgeon (NX), highly experienced in LRP, having performed more than 100 LRP previously to this study.

All patients received a standardized pre-operative evaluation including measurement of body mass index, preoperative serum prostate specific antigen (PSA), transrectal ultrasound of the prostate for prostate volume, digital rectum examination (DRE), prostate magnetic resonance imaging (MRI) and ultrasound guided transrectal prostate biopsy. In selected cases (total PSA level>10ng/mL and/or Gleason score≥7, or clinical T3 prostate cancer), we also obtained a bone scan. Patient's characteristics are shown in Table-1. Baseline urinary symptoms were measured using the IPSS and the Expand Prostate Cancer Index Composite validated health-related QOL (HRQOL) instruments.

**Table 1:** patient characteristics and perioperative parameters.

		Group A	Group B	P value
Patient, n		79	82	
Age, yr		67.88±6.65	65.79±7.27	0.08
BMI, kg/m2		25.35±2.13	24.26±2.88	0.77
Preoperative PSA, ng/mL		26.86±31.95	33.07±40.65	0.13
Prostate weight, g		41.92±14.76	42.06±19.34	0.24
IPSS score, n		6 (0~19)	7 (0~18)	0.79
Biopsy Gleason score, n (%)				0.42
	≤6	45.8%	82.9%	
	7	45.8%	35.1%	
	≥8	8.5%	7.0%	
Operative time, min (range)		130.81±21.66	147.33±29.89	0.001
Estimated blood loss, mL		225.42+164.96	232.63±217.93	0.38
Transfusion rate (%)		(3/79) 3.79%	(5/82) 6.09%	0.50
Duration of Catheter, d		16.13±16.47	13.96±2.20	0.18
Nerve-sparing procedure, n (%)				0.11
Bilateral nerve sparing		(16/79) 20.25%	(27/82) 32.92%	
Unilateral nerve sparing		(28/79) 35.4%	(19/82) 23.17%	
Non-nerve sparing		(35/79) 44.3%	(36/82) 43.90%	
Complications, n (%)		8/79 (10.13%)	6/82 (7.32%)	0.53
Complications grade I		7/79 (8.9%)	4/82 (4.88%)	0.32
Complications grade II		1/79 (1.3%)	2/82 (2.4%)	0.58

**SD** = standard deviation; **BMI** = body mass index; **PSA** = prostate specific antigen; **IPSS** = international prostate symptoms score

Urinary continence was assessed using the self-administrated validated Expanded Prostate Cancer Index Composite (EPIC) questionnaire at 1, 2, 4, 12, 24 and 52 weeks after catheter removal (13). The questionnaire was performed either at our outpatient or by telephone interview. The definition of continence was based on patient's responses to the items selected to reflect the range of incontinence severity. The items were (‘4 weeks’ was changed to ‘1 or 2 weeks’ for the questionnaire at 1 or 2 weeks after catheter removal): (i) Over the past 4 weeks how often have you leaked urine? (ii) Which of following best describes your urinary control during the last 4 weeks? (iii) How many pads or adult diapers per day did you usually use to control leakage? Patients were considered continent if they answered ‘Not at all’ to (i), ‘total control’ to (ii), and ‘No pads’ to (iii). Patients were considered incontinent when they were lost for follow-up.

## SURGICAL TECHNIQUE

All surgeries were performed utilizing an extraperitoneal approach and five ports technique (14). After creating a working space via an extraperitoneal approach, the prostate, bladder and endopelvic fascia were exposed. The endopelvic fascia was incised on both sides, and blunt dissection was performed proximally towards the apex of the prostate. After puboprostatic ligaments were fully dissected with cold scissors, a 2/0 Polysorb GS-22 needle was used to ligate dorsal venous complex. The bladder neck was carefully dissected and preserved followed by dissection of the seminal vesicles and incision of Denonvillier's fascia. The prostatic pedicle was clipped close to the prostate and cut with cold scissors step by step. A nerve sparing procedure was performed in patients with cT1-cT2a prostate cancer and biopsy Gleason score≤7. The prostatic fascia was incised sharply using cold scissor, 1cm above the prostate apex, and mobilized downward to the apex. Care was taken to keep segregation outside the prostatic capsule. The puboprostatic ligaments were preserved. Apical dissection of the prostate and division of the urethra were then performed. The urethra was cut at the middle between the external urethral sphincter and the apex of the prostate with cold scissors. Bilateral pelvic lymphadenectomy was performed in all patients. All specimens were removed in a retrieval endobag.

In Group-A, the urethrovesical anastomosis was performed using a 3/0 monocryl absorbable suture. The procedure was completed using one suture technique. The first suture was performed at the right posterior area of the bladder neck from outside to the inside, and placed this suture from inside to outside at the corresponding section of the urethral stump, and one knot was made. Then the procedure of outside to inside at bladder neck and inside to outside at urethral stump was repeated in the left side area of the bladder neck. An 18F Silastic Foley catheter was placed gently into the bladder. The urethrovesical anastomosis was accomplished when the suture was continued around from left side to right side of the bladder neck.

**Figure 1:**
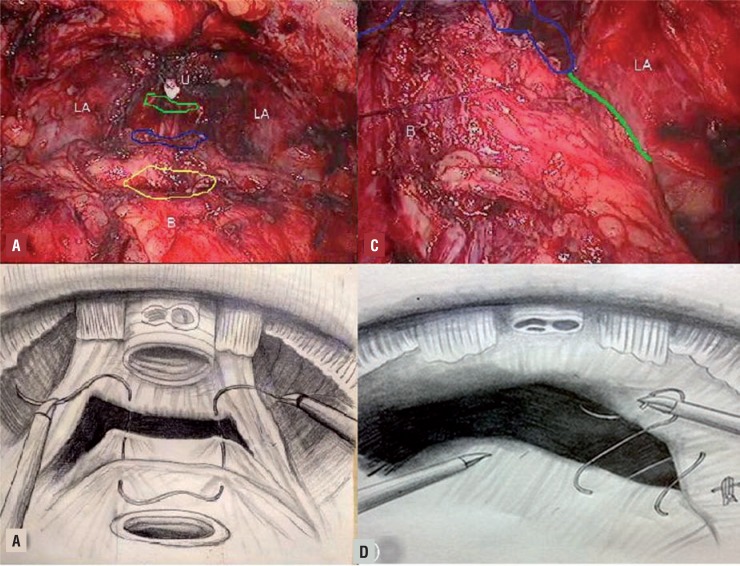
A) The first layer of reconstruction (approximating the musculofascial plate posterior to the urethra (green line region) and the Denonvillier's fascia posterior to the bladder (blue line region) and the bladder musculature. u, urethra, LA, levator ani, B, bladder neck, (the yellow region). B) Illustration shows a separate “u” type of suture that is used for the first layer reconstruction. C) A 3/0 monocryl absorbable suture is used to approximate the remaining arcus tendineus and distal triangular plate anterior to the urethra (including the residual of the endopelvic fascia and puboprostatic ligaments, rhabdosphincter, dorsal venous complex) to the bladder neck. D) Illustration shows the anterior reconstruction.

In Group-B, the total reconstruction was started with posterior reconstruction (15). Posterior reconstruction was accomplished by suturing the bladder musculature, Denonvillier's fascia and the musculofascial plate posterior to the urethra (Figures 1A and B). The second step of the reconstruction was the urethrovesical anastomosis, which was made in the manner than in Group-A. The third step was anterior reconstruction consisting of reattachment of the arcus tendineus and puboprostatic plate to the bladder neck (Figures 1C and D). A 3/0 monocryl absorbable suture was used to approximate the remaining arcus tendineus and distal triangular plate anterior to the urethra (including the residual of the endopelvic fascia and puboprostatic ligaments, rhabdosphincter, dorsal venous complex) to the bladder neck (16).

### statistical analysis

The categorical variables were summarized in frequency Tables. Continuous variables were presented as the mean±standard deviation. Two––group t-tests were used to compare numerical variables. Pearson's chi-squared test was used to compare categorical variables. Data was processed using SPSS 17.0. Statistical significance was defined as P<0.05.

## RESULTS

There was no significant difference between Group A and B regarding patient age (67.88±6.65 versus 65.79±7.27, P=0.08), BMI (25.35±2.13 versus 24.26±2.88kg/m^2^, P=0.77), preoperative serum PSA (26.86±31.95 versus 33.07±40.65ng/ml, P=0.13), prostate weight (41.92±14.76 versus 42.06±19.34g, P=0.24), IPSS (6 versus 7, P=0.79), Gleason score (P=0.42), mean estimated blood loss (225.42±164.96 versus 232.63±217.93mL, P=0.38), transfusion rates (3.79% versus 6.09%, P=0.50), nerve-sparing procedure (P=0.11) and duration of catheter (16.13±16.47 versus 13.96±2.20d, P=0.18) (Table-1). The operative time was on average 17 minutes longer in the procedure of LRP with total reconstruction technique (130.81±21.66 versus 147.33±29.89 min, P=0.001) (Table-1).

Early postoperative complications were encountered in both groups. In Group-A, 4/79 patients with anastomotic site leakage were treated with prolonged catheterization for 1 additional week. In Group-B, 4/82 patient with anastomotic site leakage were dealt with the same procedure. In Group-A, 3/79 patients with acute urinary retention after catheter removal were treated with re-catheterization for 1 week, and one patient with postoperative anastomotic stenosis was treated with urethral dilation 4 times (once per week). In Group-B, 2 patients with anastomotic stenosis were treated with urethral dilation. No significant difference was noted between the two groups with respect to anastomotic site leakage, postoperative urethral stenosis and the severity of complications (based on the Clavien-Dindo classification, Grade I and II) (Table-1) (17).

The two groups had no significant differences in their pathologic stage, positive surgical margin (PSM), or Gleason score of the surgical specimen (Table-2). The incidence of PSM at the prostate apex was 4/79 in Group-A and 7/82 in Group-B. There was no difference in the number of PSM at the prostate apex in the two groups (P=0.383).

**Table 2:** Pathologic stage and continence rates between groups.

		Group A	Group B	P value
Pathologic stage n (%)				0.089
pT0		(2/79) 2.53%	(3/82) 3.65%	
pT2a		(33/79) 41.77%	(38/82) 46.34%	
pT2b		(19/79) 24.05%	(15/82) 18.29%	
pT3a		(18/79) 22.78%	(13/82) 15.85%	
pT3b		(4/79) 5.06%	(13/82) 15.85%	
pT4		(3/79) 3.79%	(0/82) 0%	
PSM rate (%)		(10/79) 12.65%	(13/82) 15.85%	0.562
PSM at the apex		(4/79) 5.06%	(7/82) 8.53%	0.383
Gleason sore-final specimen (%)				0.246
	≤6	(27/79) 34.17%	(38/82) 46.34%	
	7	(35/79) 44.30%	(32/82) 39.02%	
	≥8	(17/79) 21.51%	(12/82) 14.63%	
**Continence rates**				
	1wk	(6/79) 7.59% %	(11/82) 13.41%	0.230
	2wk	(16/79) 20.25%	(27/82) 32.92%	0.069
	4wk	(30/79) 37.97%	(54/82) 65.85%	<0.001
	12wk	(46/79) 58.22%	(67/82) 81.71%	0.001
	24wk	(64/79) 81.01%	(74/82) 90.24%	0.094
	52wk	(71/79) 89.87%	(78/82) 95.12%	0.205

**PSM** = Positive surgical margin.

**Figure 2:**
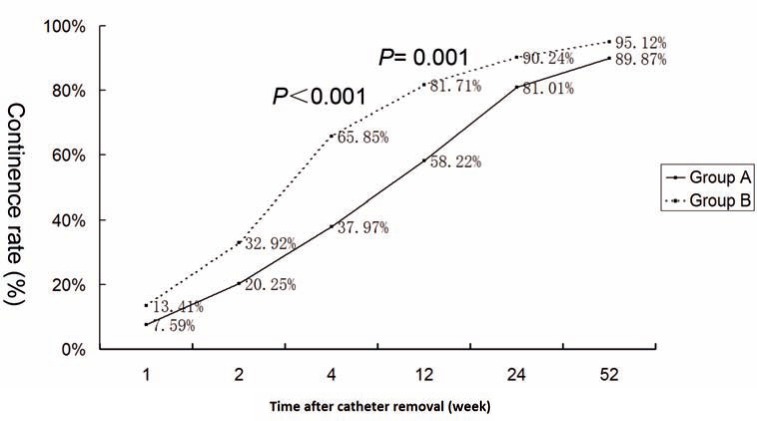
postoperative urinary continence rate of both groups.

We evaluated the questionnaires (EPIC) from 76 patients in Group A and 80 patients in Group B. Five patients (3 patients in Group A and 2 patients in Group B) were lost to follow-up, who were considered to be urinary incontinent. Group B had significantly higher continence rates at 4 and 12 weeks after catheter removal (P<0.001 and P=0.001). The continence rate of the two groups at 1, 2, 24 and 52 weeks were not significantly different (Table-2, Figure-2).

## DISCUSSION

Urinary incontinence is a major quality––of-life concern for patients undergoing radical prostatectomy. The causes of urinary incontinence after radical prostatectomy are likely multifactorial and include both functional and anatomical changes related to removal of the prostate gland and alterations in the pelvic floor musculature and the urinary sphincter complex (3, 17).

Attempts have been made to modify Walsh's anatomic radical prostatectomy to prevent injury or to reconstruct the rhabdo-urinary sphincter. Rocco et al. (18) first described post reconstruction in a study and found continence improved in the reconstruction group at 1 and 3 months. When this technique was transferred to LRP, a significant improvement was found at 1 month, but not at 3 months (15). In a well designed but not randomized trial including 803 patients, Coelho et al. (19) showed that post reconstruction shortened continence recovery time and improved early continence in patients undergoing robot-assisted radical prostectomy.

Tewari and coworkers (16) evaluated groups of patients undergoing anterior reconstruction alone, anterior and posterior reconstruction, and a historical control Group. Both types of reconstruction were significantly better in terms of time to continence. In their study, multiple interrupted sutures were used to reapproximate the posterior urethral plate and a running suture was used to reapproximate the arcus tendineus and puboprostatic plate to the bladder neck. Of note, a prospective randomized study by Menon in 2008 found no difference in early continence in patients undergoing robot-assisted radical prostatectomy with periprostatic tissue reconstruction and patients undergoing a standard single-layer anastomosis (20). Hoshi et al. (21) reported that the total pelvic floor reconstruction technique during laparoscopic radical prostatectomy improved the postoperative 3, 6 and 12 months urinary continence outcomes. The total pelvic floor reconstruction technique included two concepts involving posterior and anterior reconstructions. In posterior reconstruction, Denonvillier's fascia was approximated to the bladder neck and the median dorsal raphe by slipknot. The anterior surface of the bladder-neck was approximated to the anterior detrusor apron and the puboprostatic ligament collar for anterior reconstruction.

After the prostate specimen was removed, we performed the total reconstruction technique for patients in Group B. The first layer of the anastomosis was the posterior reconstruction. A separate “U” type of suture was used for the entire anastomosis. This was based on the principles of Rocco's posterior reconstruction technique. But, unlike his technique, we performed a single-layer reconstruction, approximating the musculofascial plate posterior to the urethra, the Denonvillier's fascia posterior to the bladder and the bladder musculature. This modified posterior reconstruction technique was to provide support to the urethra, restore it to a more anatomic position and facilitate the tension-free approximation of bladder neck to the urethral stump (Figures 1A and B). During the procedure of posterior reconstruction, we decreased the pressure of CO_2_ to 8mmHg, so it was easy to approximate the bladder neck to the urethra. Then the pressure was increased to 20mmHg to make space for the urethrovesical anastomosis. When the posterior wall of the bladder neck and urethra stump were sutured, the pressure decreased to 15mmHg.

We performed an anterior reconstruction after the urethrovesical anastomosis. Our anterior reconstructive technique was modified from the Tewari technique. The third-layer of reconstruction was performed by suturing the arcus tendineus and distal triangular plate anterior to the urethra to the anterior musculofascial of bladder neck, which was different from the anterior reconstruction technique described by Hoshi. This technique allowed the anatomic structure of the bladder neck and urethra to be reconstructed as much as possible (Figures 1C and D).

We found that patients who underwent total reconstructive repair had significantly improved urinary control at 4 and 12 weeks compared to those undergoing a standard LRP (Figure-2). Total reconstruction provides anatomical support to the urethra, and stabilizes the urethra and striated sphincter in the normal anatomical position. The posterior reconstruction enabled a tension free anastomosis and recreated the posterior support for the sphincter. The complication rates were similar in the total reconstruction and control Groups (P=0.53). Moreover, our study concluded that the total reconstruction technique was not associated with a higher incidence of positive surgical margin (PSM) (12.65% versus 15.85%, P=0.562).

There were some flaws in this study. It was a retrospective case-controlled study rather than a prospective randomized trial. Another limitation was that 5 patients in our study were lost to follow-up. We evaluated these patients as all in-continent and found that the findings are still significant at 4 and 12weeks (P=0.002 and P=0.001). The third limitation was the longer term maintenance of continence could not be evaluated. Urodynamics were not performed in this study to evaluate bladder stability and its contribution to continence. The fourth limitation was the techniques employed in the study were sequential, thus patients from Group B may have benefited from increased surgeon experience since they were treated sequentially to patients from Group A.

## CONCLUSIONS

We employed a total reconstruction technique supporting the posterior and anterior structure of urethra, which improved early continence of patients undergoing laparoscopic radical prostatectomy in our study. This technique was easy and simple to perform, and resulted in a significant improvement in early continence at 4 and 12 weeks. Our findings support the need for further studies on technical refinements for earlier urinary continence in LRP.
